# Comparing routine inpatient data and death records as a means of identifying children and young people with life-limiting conditions

**DOI:** 10.1177/0269216317728432

**Published:** 2017-08-29

**Authors:** Stuart Jarvis, Lorna K Fraser

**Affiliations:** Department of Health Sciences, University of York, York, UK

**Keywords:** Palliative medicine, child, inpatients, cause of death

## Abstract

**Background:**

Recent estimates of the number of children and young people with life-limiting conditions derived from routine inpatient data are higher than earlier estimates using death record data.

**Aim:**

To compare routine inpatient data and death records as means of identifying life-limiting conditions in children and young people.

**Design:**

Two national cohorts of children and young people with a life-limiting condition (primary cohort from England with a comparator cohort from Scotland) were identified using linked routinely collected healthcare and administrative data.

**Participants:**

A total of 37,563 children and young people with a life-limiting condition in England who died between 1 April 2001 and 30 March 2015 and 2249 children and young people with a life-limiting condition in Scotland who died between 1 April 2003 and 30 March 2014.

**Results:**

In England, 16,642 (57%) non-neonatal cohort members had a life-limiting condition recorded as the underlying cause of death; 3364 (12%) had a life-limiting condition-related condition recorded as the underlying cause and 3435 (12%) had life-limiting conditions recorded only among contributing causes. In all, 5651 (19%) non-neonates and 3443 (41%) neonates had no indication of a life-limiting condition recorded in their death records. Similar results were seen in Scotland (overall, 16% had no indication of life-limiting conditions). In both cohorts, the recording of life-limiting condition was highest among those with haematology or oncology diagnoses and lowest for genitourinary and gastrointestinal diagnoses.

**Conclusion:**

Using death record data alone to identify children and young people with life-limiting condition – and therefore those who would require palliative care services – would underestimate the numbers. This underestimation varies by age, deprivation, ethnicity and diagnostic group.

**What is already known about the topic?**

- Children and young people – with life-limiting conditions have complex healthcare needs – often with repeated hospital admissions, particularly at end of life.
- Recent estimates of prevalence of life-limiting conditions among children and young people using routinely collected inpatient data are much higher than earlier estimates using death records.

**What this paper adds?**

- Compares identification of life-limiting conditions in children and young people using death records and inpatient data in the same population for the first time.
- Shows where the differences occur (by diagnostic group, age group, ethnic group and deprivation).
- Identifies shortcomings in use of death records to identify life-limiting conditions in children and young people.

**Implications for practice, theory or policy**

- Inpatient-based estimates of life-limiting conditions prevalence among children and young people should be used for service planning.
- Epidemiological studies based on life-limiting conditions identification from death record data may be biased.
- Use of death records for life-limiting conditions identification is particularly limited in countries that only record the underlying cause of death.

## Background

Children and young people with life-limiting conditions (encompassing both life-limiting conditions that will lead to premature death, for example, Duchenne muscular dystrophy, and life-threatening conditions that may lead to premature death, but may be cured, for example, cancer) typically have complex healthcare needs, often with repeated hospital admissions, particularly towards the end of life.^1,2^ Planning paediatric palliative care services, where there is evidence that demand outstrips provision,^3^ requires accurate estimates of prevalence (in addition to estimates of future survival times).

Recent prevalence estimates^3–5^ of children and young people with a life-limiting condition in England and Scotland, which used an International Classification of Diseases Version 10^6^ coding framework to identify life-limiting conditions within routine inpatient hospital data, were much higher than earlier estimates.^7–9^ This may be due to previous estimates being based on death record data, about which there are concerns of quality and completeness,^9^ but no work has been previously published comparing the two methods within the same population to quantify these differences. Previous research has found that there are discrepancies between recorded cause of death and conditions recorded during life for a population sample^10^ and for children with chronic conditions,^11^ but these differ from this study in not focusing on children (first study) and not limiting analyses to life-limiting conditions (both studies). In the latter study, it was noted that the discrepancies may be due to chronic conditions not being related to the cause of death, but for children with life-limiting conditions most deaths are expected to be related to the condition.

This study compares methods of identifying children and young people with a life-limiting condition by analysing recorded cause of death for children and young people identified with a life-limiting condition from routinely collected English and Scottish inpatient hospital data.

## Methods

### Definition of life-limiting conditions

Individuals with a life-limiting condition were identified using a refined version^5^ of a previously developed International Classification of Diseases Version 10 coding framework.^4^

### Data used and cohort identification

Two national cohorts were identified: a primary cohort from England and a comparison cohort from Scotland (the latter was used to assess whether any differences found were unique to England and is described in the supplement).

For England, linked inpatient Hospital Episode Statistics data and Office for National Statistics (ONS) death records were used. Data access was granted by the Health and Social Care Information Centre and ONS microdata release panel (ref: NIC-379681-D6L7G). Children and young people with a life-limiting condition were identified by matching recorded diagnostic codes in inpatient records against the coding framework, for individuals aged 0–25 in the English study period (1 April 2001 to 30 March 2015). The cohort was restricted to individuals with a death record with a date of death in the respective study period.

### Data management

The English datasets were linked by the Health and Social Care Information Centre based on National Health Service number, gender, date of birth and postcode.^12^ Management of Scottish datasets is described in the supplement.

Date of birth was assigned as the most commonly recorded date in the inpatient data. Dates of death came from death records. Individuals with invalid dates of death (more than 1 day before the beginning of an inpatient record) were excluded from the cohort.

Individuals who had died were assigned an age group at death: neonates (0–27 days), post-neonatal infants (28–364 days), 1–5, 6–10, 11–15, 16–20, 21–25 and over 25 years. Age at death was determined by subtracting date of birth from date of death. Only month and year of birth was provided, so post-neonatal ages of death were approximated setting all birth dates to the 15th day of the month. Neonates were separately identified from the presence of neonate-specific cause of death fields in the death records.

Published populations^13^ and Index of Multiple deprivation 2004^14^ rankings^15^ for the Lower Super Output Areas provided in the data were used to assign each individual to one of five Index of Multiple Deprivation categories, with approximately 20% of the population of England in each category. The Index of Multiple Deprivation 2004 is an area-based measure of deprivation under seven domains.^16^ Individuals were assigned the last recorded category before death.

Recorded ethnic categories were collapsed to seven groups: White, Indian, Pakistani, Bangladeshi, Black, Mixed and Other. The most commonly recorded ethnicity (from the collapsed groupings) was assigned to each individual.

Life-limiting condition diagnoses were categorised into 11 groups based on the main International Classification of Diseases Version 10 chapters: neurology, haematology, oncology, metabolic, respiratory, circulatory, gastrointestinal, genitourinary, perinatal, congenital and ‘other’. Individuals may be assigned more than one diagnostic group if they had more than one life-limiting condition recorded in the inpatient hospital data. A primary diagnostic group, the most common diagnostic group across all inpatient records, was assigned to each individual. Where there was more than one most common diagnostic group, later diagnoses were prioritised (diagnoses from the earliest records were progressively removed until the tie was broken).

### Analyses

#### Cause of death

The English death records contained underlying cause of death (the condition that initiated the chain of events that led to death, not necessarily the proximate cause of death) and other causes of death for non-neonates. It was expected that most deaths in the cohort (all of whom were known to have a life-limiting condition) would have a life-limiting condition as the underlying cause. The proximate cause of death may differ – for example, it may be an infection – but with a life-limiting condition as the underlying cause in most cases, making infection either more likely or more severe. A small number of individuals were also expected to die from trauma (e.g. car or other accidents) not related to the life-limiting condition. Whether the underlying cause of death was a life-limiting condition was checked using the coding framework. If not a life-limiting condition, underlying cause was assessed to see whether it was related to a life-limiting condition identified in the inpatient data. For example, nonspecific cerebral palsy as cause of death was considered related to quadriplegic cerebral palsy; unspecified congenital malformations of heart to tetralogy of Fallot. Finally, for those with underlying cause, neither a life-limiting condition nor life-limiting condition-related, contributing causes of death were checked against the life-limiting condition coding framework. Where life-limiting condition was recorded as a contributing cause, trauma-related underlying causes were determined (all codes starting S; T0; T1; T2; T30; T31; T32; T5; T6; T7; T9; V; W; X; Y1; Y2; Y3) as it was expected that most deaths in the cohort not due to life-limiting conditions would be due to trauma.

For English neonate death records, underlying cause of death was not specified. All causes of death were checked for life-limiting conditions or being life-limiting condition-related (only presence or absence of life-limiting conditions among causes of death could be determined, subdivision into underlying, related or contributory causes was not possible for this age group).

The analyses were split by age group (at death), by ethnic group, by deprivation category, by diagnostic category and by groups of financial year of death. Neonates were excluded from analyses by ethnic group, deprivation category, diagnostic category and year as they could not be categorised as having underlying, related or contributory cause of death as a life-limiting condition.

#### Statistical modelling

Predictors of life-limiting conditions being present in death records were explored. A binary outcome variable was defined indicating the presence of life-limiting conditions in a death record, set to 1 if the underlying cause was a life-limiting condition or was related to a life-limiting condition or a contributing cause of death was a life-limiting condition and to 0 if there was no indication of life-limiting conditions. Candidate predictor variables were age group at death, primary diagnostic group, deprivation category and ethnic group. Multivariable logistic regression models were fitted, with candidate predictors added in turn and retained if their odds ratios were significantly (*p* < 0.05) different to 1 or inclusion reduced Schwarz’s Bayesian Information Criterion for the model by more than 2.^17,18^ Interactions between deprivation and ethnic group were also considered (using the same inclusion criteria). Models stratified between neonates and non-neonates and between oncology and non-oncology primary diagnoses were also developed as it was observed that levels of life-limiting condition recording in death records varied greatly between these groups. Individuals with data missing for any included predictors were excluded from the corresponding models.

## Results

### England

#### Cohort size

A total of 411,154 children and young people with a life-limiting condition were identified between 1 April 2001 and 31 March 2015 while aged 0–25; 37,784 had death records with a date of death in the period. In all, 221 death records (0.6%) were considered invalid as there were one or more inpatient admissions after their recorded date of death and were excluded, leaving 37,563 individuals in the final cohort. There were 73 individuals (0.2% of final cohort) with conflicting dates of birth between records: in each case, the more commonly recorded date of birth was used. Numbers of deaths in groups of years and cohort demographics are shown in Table 1.

**Table 1.** Demographics, missing data and cause of death by year of death for the English cohort.

		Financial year of death		
		2001/2002–2004/2005	2005/2006–2009/2010	2010/2011–2014/2015
Deaths in period		9055	13,984	14,524
Deaths by age group				
Neonate		1781	3118	3572
Post-neonatal infant		1476	2282	2161
1–5 years		1334	1793	1723
6–10 years		713	928	891
11–15 years		882	1120	886
16–20 years		1233	1697	1465
21–25 years		1324	2021	2009
Over 25 years		312	1025	1817
Deaths by ethnic group				
White		5837	9505	10,154
Indian		199	359	381
Pakistani		478	995	1089
Bangladeshi		102	223	195
Black		348	739	851
Mixed		84	300	383
Other		358	641	730
Not known		1649	1222	741
Not known (excluding neonates)		964	622	299
Deaths by deprivation category				
1 (most deprived)		2496	3912	4157
2		1716	2802	2969
3		1507	2123	2309
4		1325	1889	2003
5 (least deprived)		1200	1757	1704
Not known		811	1501	1382
Not known (excluding neonates)		221	390	310
Deaths by diagnostic category				
Neurology		1530	2698	2993
Haematology		1486	2258	2417
Oncology		2670	3431	3433
Respiratory		1263	2268	2975
Circulatory		675	1267	1242
Gastrointestinal		496	931	1272
Genitourinary		993	1907	2493
Perinatal		1447	2673	3328
Congenital		2286	3830	4190
Metabolic		413	791	945
Other		205	336	429
Life-limiting condition recording (excluding neonates)				
As underlying cause	Matched^a^	4496 (62%)	6204 (57%)	5942 (54%)
	Related^a^	759 (10%)	1275 (12%)	1330 (12%)
As contributing cause		780 (11%)	1319 (12%)	1336 (12%)
With trauma-related underlying cause		26	46	44
Not recorded		1239 (17%)	2068 (19%)	2344 (21%)
All non-neonate deaths		7274	10,866	10,952

a ‘Matched’ underlying causes are those that matched diagnostic codes within the life-limiting condition coding framework. ‘Related’ causes were considered to be related to framework diagnoses present for the individual in the inpatient data.

#### Missing data

In total, 6% of non-neonates had unknown ethnicity, although this figure reduced to 3% for individuals with a date of death on or after 1 April 2009. Including neonates, 10% missed ethnicity information (5% excluding deaths before 1 April 2009, Table 1). In all, 3% of non-neonates had an unknown deprivation category, rising to 10% including neonates. There were no missing data for age group at death or diagnostic category. To test for effects of missing data, a sensitivity analysis was undertaken in the statistical modelling, generating models for the whole time period and also for only 1 April 2009 onwards and with and without neonates.

#### Cause of death

Among non-neonates, 16,642 (57%) had a life-limiting condition recorded as underlying cause of death (Table 2); 3364 (12%) had a life-limiting condition-related underlying cause and 3435 (12%) had life-limiting conditions only among contributing causes, of which 116 had a trauma-related underlying cause (Table 2). In total, 5651 (19%) had no indication of life-limiting conditions in their death records. Among neonates, 5028 (59%) had a life-limiting condition or life-limiting condition-related condition among their causes of death; 3443 (41%) had no indication of life-limiting conditions among causes of death.

**Table 2.** Recorded cause of death for cohort members, split by age group at death.

Life-limiting condition recording		Age at death – English data								
		Neonate	Post-neonatal infant	1–5 years	6–10 years	11–15 years	16–20 years	21–25 years	>25 years	All non-neonates
As underlying cause	Matched^a^	5028^b^ (59%)	2463 (42%)	2598 (54%)	1601 (63%)	1865 (65%)	2808 (64%)	3378 (63%)	1929 (61%)	16,642 (57%)
	Related^a^		1231 (21%)	673 (14%)	261 (10%)	302 (10%)	386 (9%)	300 (6%)	211 (7%)	3364 (12%)
As contributing cause			903 (15%)	631 (13%)	278 (11%)	323 (11%)	433 (10%)	522 (10%)	345 (11%)	3435 (12%)
With trauma-related underlying cause			<10	<10	<10	14	21	43	20	116
Not recorded		3443 (41%)	1322 (22%)	948 (20%)	392 (15%)	398 (14%)	768 (17%)	1154 (22%)	669 (21%)	5651 (19%)
All deaths in age group		8471	5919	4850	2532	2888	4395	5354	3154	29,092

a ‘Matched’ underlying causes are those that matched diagnostic codes within the life-limiting condition coding framework. ‘Related’ causes were considered to be related to framework diagnoses present for the individual in the inpatient data.

b Neonate cause of death could not be split between underlying, related and contributory life-limiting condition in the English data – all causes of death that were a life-limiting condition or related to a life-limiting condition were counted.

##### Cause of death by financial year of death

There was only minor variation in recording of life-limiting conditions across financial year of death (Table 1). The proportion of deaths reporting a life-limiting condition as underlying cause varied from 54% to 62% while the proportion with no indication of life-limiting conditions varied from 17% to 21%. There was no clear trend over time.

##### Cause of death by age at death

Neonates were significantly more likely to have no indication of life-limiting conditions in death records than non-neonates (40.6%, 95% confidence interval (CI): 39.6%–41.7% compared to 19.4%, 95% CI: 19.0%–19.9%) (Table 2). Recording of a life-limiting condition as underlying cause of death was lowest (2463, 42%) among post-neonatal infants, but they had the highest percentage of life-limiting condition-related deaths (1231, 21%). The youngest and eldest were most likely among the age groups to have no indication of life-limiting condition in death records.

##### Cause of death by ethnic group

Children and young people of Bangladeshi or Black ethnicity were less likely than White children and young people to have a life-limiting condition as underlying cause of death (Bangladeshi: 48.5%, 95% CI: 43.5%–53.4%; Black: 49.4, 95% CI: 46.9%–52.0%; White: 58.9%, 95% CI: 58.2%–59.6%) although they had higher levels of life-limiting condition-related underlying or contributory causes of death (Table 3). Black children and young people were significantly more likely than White children and young people to have no indication of life-limiting conditions in their death records (Black: 24.0%, 95% CI: 21.8%–26.2%; White: 18.8%, 95% CI: 18.3%–19.3%).

**Table 3.** Recorded cause of death for England cohort members, split by ethnic group.

Life-limiting condition recording		Ethnic group – English data								
		White	Indian	Pakistani	Bangladeshi	Black	Mixed	Other	Unknown	All groups
As underlying cause	Matched^a^	12176 (59%)	415 (57%)	1104 (53%)	191 (48%)	729 (49%)	299 (54%)	749 (56%)	979 (52%)	16,642 (57%)
	Related^a^	2325 (11%)	69 (9%)	263 (13%)	50 (13%)	201 (14%)	74 (13%)	170 (13%)	212 (11%)	3364 (12%)
As contributing cause		2284 (11%)	100 (14%)	324 (16%)	74 (19%)	191 (13%)	68 (12%)	186 (14%)	208 (11%)	3435 (12%)
With trauma-related underlying cause		92	⩽10	⩽10	⩽10	⩽10	⩽10	⩽10	⩽10	116
Not recorded		3883 (19%)	144 (20%)	376 (18%)	79 (20%)	354 (24%)	108 (20%)	221 (17%)	486 (26%)	5651 (19%)
All deaths associated with ethnic group		20668	728	2067	394	1475	549	1326	1885	29,092

a ‘Matched’ underlying causes are those that matched diagnostic codes within the life-limiting condition coding framework. ‘Related’ causes were considered to be related to framework diagnoses present for the individual in the inpatient data.

##### Cause of death by deprivation category

Individuals in the most deprived categories were less likely than those in the least deprived category to have a life-limiting condition recorded as the underlying cause of death (category 1: 53.7%, 95% CI: 52.6%–54.7%; category 5: 62.6%, 95% CI: 61.1%–64.1%) and more likely to have no indication of life-limiting conditions (category 1: 22.3%, 95% CI: 21.5%–23.2%; category 5: 15.7%, 95% CI: 14.5%–16.8%) (Table 4).

**Table 4.** Recorded cause of death for cohort members, excluding English neonates, split by deprivation category of last recorded deprivation score.

Life-limiting condition recording		Deprivation category – English data				
		1 (most deprived)	2	3	4	5 (least deprived)
As underlying cause	Matched^a^	4639 (54%)	3436 (56%)	3012 (59%)	2576 (60%)	2494 (63%)
	Related^a^	948 (11%)	727 (12%)	605 (12%)	510 (12%)	446 (11%)
As contributing cause		1125 (13%)	767 (12%)	563 (11%)	449 (11%)	418 (10%)
With trauma-related underlying cause		34	30	21	18	11
Not recorded		1930 (22%)	1259 (20%)	906 (18%)	737 (17%)	624 (16%)
All deaths associated with deprivation category		8642	6189	5086	4272	3982

The categories are population weighted so that 20% of the general population is in each category.

a ‘Matched’ underlying causes are those that matched diagnostic codes within the life-limiting condition coding framework. ‘Related’ causes were considered to be related to framework diagnoses present for the individual in the inpatient data.

##### Cause of death by diagnostic group

In all, 94% of individuals with an Oncology diagnosis had a life-limiting condition as underlying cause of death; only 3% had no indication of life-limiting conditions in their death records (Table 5). Only 28% of patients with a perinatal diagnosis had a life-limiting condition as the underlying cause of death, while those with genitourinary diagnoses were most likely (31%) to have no life-limiting condition among any cause of death.

**Table 5.** Recorded cause of death for cohort members, excluding English neonates, split by diagnostic group.

Life-limiting condition recording		Diagnostic group – English data										
		Neurology	Haematology	Oncology	Respiratory	Circulatory	Gastrointestinal	Genitourinary	Perinatal	Congenital	Metabolic	Other
As underlying cause	Matched^a^	3143 (45%)	5105 (83%)	8862 (94%)	3188 (49%)	1457 (52%)	1242 50%)	2167 (43%)	652 (28%)	2766 (41%)	1288 (65%)	700 (72%)
	Related^a^	1433 (20%)	108 (2%)	22 (0%)	100 (12%)	788 (16%)	443 (7%)	174 (8%)	411 (28%)	656 (24%)	1638 (5%)	25 (2%)
As contributing cause		946 (13%)	457 (7%)	292 (3%)	1046 (16%)	431 (15%)	425 (17%)	900 (18%)	400 (17%)	1088 (16%)	333 (17%)	155 (16%)
With trauma-related underlying cause		33	<10	11	18	<10	16	32	<10	29	<10	<10
Not recorded		1516 (21%)	455 (7%)	299 (3%)	1448 (22%)	455 (16%)	650 (26%)	1571 (31%)	609 (26%)	1334 (20%)	1983 (13%)	91 (9%)
All deaths associated with diagnostic group		7028	6125	9474	6467	2782	2491	5046	2319	6823	1983	970

a ‘Matched’ underlying causes are those that matched diagnostic codes within the life-limiting condition coding framework. ‘Related’ causes were considered to be related to framework diagnoses present for the individual in the inpatient data.

#### Multivariable model

The final model (Table 6) showed some differences to the univariable analyses. Neonates were least likely to have a life-limiting condition recorded, but 21–25 year olds, and 1–5 and 6–10 year olds were next least likely to have a life-limiting condition recorded (odds ratio compared to post-neonatal infants: neonate 0.54, 95% CI: 0.49–0.60; 1–5 year olds 0.74, 95% CI: 0.67–0.83; 6–10 year olds 0.74, 95% CI: 0.64–0.86; 21–25 year olds 0.62, 95% CI: 0.55–0.69). Variations by ethnic group were also different in the multivariable model, with minority ethnic groups either not significantly different to White children and young people in likelihood of having a life-limiting condition recorded or more likely (Pakistani: 1.40, 95% CI: 1.25–1.57 times more likely than White children and young people; Other ethnicity 1.25, 95% CI: 1.08–1.43 times more likely than White children and young people). Children and young people in less deprived categories were more likely than children and young people in more deprived categories to have a life-limiting condition recorded (odds ratio for least deprived category 1.19, 95% CI: 1.08–1.32 compared to most deprived category). However, there were only small differences between the three least deprived categories. Primary diagnostic group showed similar patterns to those seen in the univariable analyses: haematology and oncology diagnoses were most likely to be associated with life-limiting condition recording in death records and genitourinary, gastrointestinal and perinatal diagnoses least likely.

**Table 6.** Logistic regression model, using the English data, for the odds ratio of a death record containing any indication of life-limiting condition (underlying, related or contributing) depending on age group at death, ethnic group, deprivation category and primary diagnostic group.

	Odds ratio for life-limiting condition in death record	95% confidence interval		*p* value
Age group at death				
Neonate	0.54	0.49	0.60	<0.01
Post-neonatal infant	1 (ref)			
1–5	0.74	0.67	0.83	<0.01
6–10	0.74	0.64	0.86	<0.01
11–15	0.98	0.85	1.13	0.77
16–20	0.80	0.71	0.90	<0.01
21–25	0.62	0.55	0.69	<0.01
>25	0.74	0.65	0.84	<0.01
Ethnic group				
White	1 (ref)			
Indian	1.13	0.94	1.34	0.19
Pakistani	1.40	1.25	1.57	<0.01
Bangladeshi	1.15	0.91	1.45	0.24
Black	0.99	0.87	1.11	0.81
Mixed	1.10	0.90	1.33	0.35
Other	1.25	1.08	1.43	<0.01
Last recorded deprivation category				
1 (most deprived)	1 (ref)			
2	1.11	1.02	1.20	0.01
3	1.17	1.07	1.28	<0.01
4	1.21	1.10	1.33	<0.01
5 (least deprived)	1.19	1.08	1.32	<0.01
Primary diagnostic group				
Neurology	0.09	0.08	0.10	<0.01
Haematology	0.06	0.05	0.08	<0.01
Oncology	1 (ref)			
Respiratory	0.06	0.05	0.07	<0.01
Circulatory	0.09	0.07	0.11	<0.01
Gastrointestinal	0.03	0.03	0.04	<0.01
Genitourinary	0.03	0.02	0.03	<0.01
Perinatal	0.04	0.03	0.05	<0.01
Congenital	0.10	0.08	0.11	<0.01
Metabolic	0.18	0.14	0.22	<0.01
Other	0.08	0.05	0.12	<0.01
Model characteristics				
Log likelihood	−13,744			
Bayesian information criterion	27,778			
Degrees of freedom	28			

There were only minor differences between models stratified between neonates and non-neonates or between individuals with and without oncology as primary diagnostic group. Neither was there significant evidence for interaction between deprivation and ethnic group, so the interaction was not included in the final model. The sensitivity analyses produced models that were not significantly different to the main model (Tables S7 and S8, in the supplement).

### Scotland

Full results are presented in the supplement. Of 2249 individuals in the final cohort, 57% had a life-limiting condition recorded as underlying cause of death, 14% had a life-limiting condition-related underlying cause, 12% had a life-limiting condition only among contributing causes and 16% had no indication of life-limiting conditions. Under 1 and over 20 year olds were most likely to have no life-limiting condition recorded. Those in the most deprived category were more likely (20%) to have no life-limiting condition recorded than those in the least deprived category (15%). Only primary diagnostic group showed significant associations with life-limiting condition recording in the multivariable model, with life-limiting condition recording most likely for individuals in the haematology and oncology primary diagnostic group and least likely for those in the genitourinary group (odds ratio 0.02, 95% CI: 0.01–0.05 compared to haematology and oncology).

## Discussion

This study has shown that using death certificate data alone to identify the numbers of children and young people with a life-limiting condition would have resulted in underestimation of approximately 24% when compared to those identified via inpatient hospital data (for neonates and non-neonates, counting life-limiting conditions in all cause of death fields in the English data). The Scottish data provided similar results (see supplement), although recording of life-limiting conditions was higher (only 16% had no indication of life-limiting conditions in any cause of death field). Readily available death register data in many countries only include the underlying cause of death and using these alone would underestimate the number of children and young people with a life-limiting condition further (in the English data, 31% of non-neonate cohort members had neither a life-limiting condition nor life-limiting condition-related underlying cause of death recorded; for Scotland this figure was 28% for all ages). This may explain the differences in life-limiting condition prevalence estimates between studies using routine inpatient hospital data^3–5^ and studies using death records.^7–9^

There are grounds to favour estimates from the inpatient data over estimates from the death records. The number of deaths each year among children identified with a life-limiting condition from the inpatient data (e.g. 1766 among 0–14 year olds in 2013) is close to a previous estimate of 50% of child deaths being due to life-limiting conditions^19^ (3631 0–14 year olds died in England in 2013).^20^ It would be expected that most deaths in individuals identified as having a life-limiting condition should either have the life-limiting condition as underlying cause or be due to trauma (as broadly defined here), but only 116 of the 3435 deaths with life-limiting conditions recorded as a contributing cause were trauma-related. This suggests there are quality or completeness issues with the death records. The cause of death data are produced by automated analysis of death certificates^21^ and the ONS^22^ have noted issues and changes in the way that underlying cause of death is determined in recent years, which may result in more maternal conditions as underlying cause of death being recorded as perinatal conditions.^23^ This study was concerned with whether death records for children and young people known (from routine hospital data) to have had a life-limiting condition would also record the life-limiting condition, whether as underlying or any cause of death as this affects the reliability of life-limiting condition prevalence estimates based on these data. It is immaterial, when using these data to estimate prevalence, whether any errors in recording are in the manual completion of death certificates or their later automated analysis.

As paediatric palliative care is recommended to start at the point of diagnosis (or recognition) of a life-limiting condition rather than just end-of-life care, only counting the number of children who have died from death records cannot provide a useful estimate of paediatric palliative care need. As treatments improve and survival times for many life-limiting conditions increase, death rates are likely to lag behind prevalence increases and underestimate current life-limiting condition prevalence.^9^ There is no indication that the gap between life-limiting conditions recorded on death records and indicated in inpatient data is decreasing over time, for either the English or Scottish data.

The multivariable model for England is broadly consistent with the univariable descriptive analyses – similar variations are seen for age, deprivation category and diagnostic group. Variation by ethnic group, however, appears reversed. After controlling for age, primary diagnostic group and deprivation category, individuals of Pakistani and Other ethnicity appear more likely than White individuals to have life-limiting condition recorded on their death records; there were no significant differences between White individuals and those from Indian, Bangladeshi, Black or Mixed ethnic groups. This may suggest that the previously observed variation by ethnicity was due to factors such as deprivation or diagnoses (both known to vary by ethnic group^24–27^). The decreased likelihood of life-limiting condition recording in death certificates for individuals from more deprived areas may suggest geographical variations in cause of death recording, possibly due to differences in resource provision and quality of recording. Lower likelihood of life-limiting condition recording in death records for the very young may be due to greater uncertainty about the exact causes of death; for the older groups, reduced recording may be due to death following a longer and more complex chain of events from underlying life-limiting condition to proximate cause of death, with the underlying life-limiting condition not always being recorded. Differences in life-limiting condition recording in different diagnostic categories may also be linked to the directness or otherwise of the life-limiting condition leading to death. Differences in conditions and the clarity of any link between the life-limiting condition and death in those conditions may influence levels of recording among ethnic groups. Further work is needed to investigate these issues. Similar results were seen for the multivariable model for the Scottish data (see Table S6, supplement), although most of the variations – except by primary diagnostic group – were not statistically significant, possibly due to the smaller sample size.

Underestimation of life-limiting condition prevalence from death records particularly affects some diagnostic groups (e.g. genitourinary diagnoses), the more deprived and (perhaps as a consequence of diagnostic and deprivation variations) some minority ethnic groups. This has implications for service planning where it could lead to under-provision for these groups or incorrect prioritisation of other groups that appear to have comparatively higher demand and for epidemiological studies, such as those looking at levels of particular life-limiting conditions in populations, where bias may be introduced, underestimating prevalence of some conditions more than others. For example, L.K.F.’s earlier study^4^ on prevalence of life-limiting conditions among children and young people in England would be expected to produce different estimates with different relative levels across diagnostic and age groups if using death records. Estimation of demand for service planning should be based on routine hospital data as this is both more up to date and more complete than the death records.

This study used high-quality national healthcare data and compared data independently collected in England and Scotland to verify that the variations seen were not unique to one country. The cohorts were identified using an objectively applied coding framework. However, decisions over what constituted a life-limiting condition-related underlying cause of death were subjective. The number of cohort members with unknown ethnicity and deprivation category is a concern for the robustness of the analyses with regard to ethnicity and deprivation. The missing data could not be imputed from other fields, but a sensitivity analysis using only data from 2009 to 2010 onwards (with more complete ethnicity data, Table S7, supplement) and excluding neonates (providing more complete ethnicity and deprivation data, Table S8, supplement) supports the findings using the whole study period and all age groups.

## Conclusion

Using death record data to estimate need for paediatric palliative care services should be undertaken with caution as 19% of non-neonates (31% if using only underlying cause of death) and 41% of neonates identified using the life-limiting condition coding framework as having a life-limiting condition would have been missed. The most deprived, the youngest and oldest, Black individuals and those with genitourinary, gastrointestinal or perinatal diagnoses were most likely to be missed using death records alone.

## Supplementary Material

Supplementary material
