# Supplementary material for: Comparing routine inpatient data and death records as a means of identifying children and young people with life-limiting conditions
Source: Palliat Med. 2017 Aug 29;32(2):543–53. doi: 10.1177/0269216317728432 (PMC5788076; doi:10.1177/0269216317728432)
Supplement: Supplementary material [file 728432.pdf]

Supplement Part 1: Scotland

METHODS

Data used and cohort identification

For Scotland, the inpatient dataset (SMR01), Scottish Birth Records and General Records Office death record data were used. Data access was approved by the Privacy Advisory Committee (ref: XRB14010). The Scottish data were analysed within the NSS Electronic Data Research and Innovation Service safe haven[1] and results underwent disclosure control.[2]

Children and young people with a life limiting condition were identified by matching recorded diagnostic codes in inpatient records and birth records against the International Classification of Diseases Version 10 coding framework, for individuals aged 0-25 in the study period (1 April 2003 to 30 March 2014).

Data Management

The Scottish datasets were linked by NHS National Services Scotland using the Community Health Index number.[3, 4]

Date of birth was assigned as the most commonly recorded date in the inpatient and birth data. Dates of death came from death records. Individuals with invalid dates of death (more than one day before the beginning of an inpatient record) were excluded from the cohort.

Individuals who had died were assigned an age group at death: under 1 year old (in Scotland, neonates and post-neonatal infants could not be separated), 1-5, 6-10, 11-15, 16-20, 21-25 and over 25 years. Age at death was determined by subtracting date of birth from date of death.

Provided within the data was a population-weighted Scottish Index of Multiple Deprivation 2009 category, with 10% of the population in each category. These ten categories were collapsed to five for analyses. Individuals were assigned the last deprivation category recorded before death.

The available ethnic categories were collapsed to three, due to small numbers: White, South Asian (Indian, Pakistani, Bangladeshi) and Other. Ethnic group was determined as the most commonly recorded ethnicity (from these collapsed groupings) within the data.

Life limiting condition diagnoses were categorized into 9 groups based on the main diagnostic chapters: neurology, haematology and oncology, metabolic and other, respiratory, circulatory, gastrointestinal, genitourinary, perinatal and congenital (for the Scottish data, small numbers in some groups meant that haematology and oncology were combined into one group, as were metabolic and other). Individuals may be assigned more than one diagnostic group if they had more than one life limiting condition recorded in the inpatient hospital data. A primary diagnostic group, the most common diagnostic group across all inpatient records, was assigned to each individual. Where there was more than one most common diagnostic group, later diagnoses were prioritised (diagnoses from earliest records were progressively removed until the tie was broken).

Analyses

Cause of death

The Scottish death records contained underlying and other causes of death. Whether underlying cause of death was a life limiting condition was checked using the coding framework. If not a life limiting condition, underlying cause was assessed to see whether it was related to a life limiting condition identified in the inpatient data. For example, nonspecific cerebral palsy as cause of death was considered related to quadriplegic cerebral palsy; unspecified congenital malformations of heart to tetralogy of Fallot. Finally, for those with underlying cause neither a life limiting condition nor life limiting condition-related, contributing causes of death were checked against the life limiting condition coding framework. Where life limiting condition was recorded as a contributing cause, trauma-related underlying causes were determined (all codes starting S; T0; T1; T2; T30; T31; T32; T5; T6; T7; T9; V; W; X; Y1; Y2; Y3).

The analyses were split by age group (at death), by ethnic group, by deprivation category, by diagnostic category and by financial year of death.

Statistical modelling

Predictors of life limiting condition being present in death records were explored. A binary outcome variable was defined indicating presence of life limiting condition in a death record, set to 1 if the underlying cause was a life limiting condition or was related to a life limiting condition or a contributing cause of death was a life limiting condition and to 0 if there was no indication of life limiting conditions. Candidate predictor variables were: age group at death, primary diagnostic group, deprivation category and ethnic group. Multivariable logistic regression models were fitted, with candidate predictors added in turn and retained if their odds-ratios were significantly ($p < 0.05$) different to 1 or inclusion reduced Schwarz's Bayesian Information Criterion for the model by more than 2.[5, 6] Interactions between deprivation and ethnic group were also considered (using the same inclusion criteria). Individuals with data missing for any included predictors were excluded from the corresponding models.

RESULTS

Cohort size

In Scotland, 20436 children and young people with a life limiting condition were identified between 1 April 2009 and 31 March 2014 in the Scottish inpatient and birth records. Of these, 2320 had death records in the period. 71 death records were excluded as there were one or more inpatient admissions after the recorded date of death, leaving 2249 individuals in the final cohort. Numbers of deaths in each year and cohort demographics are shown in Table S1.

Missing data

There were large numbers of missing ethnicity data in the Scottish cohort, over half for those dying in the first few years of the study (Table S1). There were no missing data for age group or diagnostic group and missing data for deprivation were at or below 1%.

Table S1: Demographics, missing data and cause of death by year of death for the Scottish cohort.

		Financial year of death	
	2003/04	2004/05	2005/06
**Deaths in year**	598	843	808
**Age group**			
Under 1 year	186	217	183
1-5 years	121	97	89
6-10 years	≤50**	66	58
11-15 years	68	79	64
16-20 years	79	155	144
21-25 years	92	171	154
Over 25 years	≤10	58	116
**Ethnic group**			
White	209	381	580
South Asian	≤20**	≤20**	≤20**
Other	≤10	≤10	≤10
Not known	385	441	188
**Deprivation category**			
1 (most deprived)	159	252	208
2	117	194	192
3	115	148	152
4	107	126	133
5 (least deprived)	96	121	118
Not known	≤10	≤10	≤10
**Diagnostic category**			
Neurology	115	175	187
Haematology & Oncology	193	286	255
Respiratory	98	196	181
Circulatory	48	60	64
Gastrointestinal	30	42	66
Genitourinary	54	105	132
Perinatal	93	97	76
Congenital	151	204	200
Metabolic & Other	69	85	72
**Life limiting condition recording**			
As underlying cause — Matched*	362 (61%)	479 (57%)	450 (56%)
As underlying cause — Related*	93 (16%)	118 (14%)	108 (13%)
As contributing cause	57 (10%)	113 (13%)	98 (12%)
With trauma-related underlying cause	≤10	≤10	≤10
Not recorded	86 (14%)	133 (16%)	152 (19%)

* Matched' underlying causes are those that matched diagnostic codes within the life limiting condition coding framework. 'Related' causes were considered to be related to framework diagnoses present for the individual in the inpatient data.

** larger values censored to prevent reconstruction of smaller censored values

Cause of death

In Scotland, 1291 cohort members (57%) had a life limiting condition recorded as underlying cause of death (Table S2); 319 (14%) had a life limiting condition-related underlying cause and 268 (12%) had life limiting conditions only among contributing causes, of which 10 had a trauma-related underlying cause (Table S2). 371 (16%) had no indication of life limiting conditions in their death records.

Cause of death by financial year of death

There was only minor variation in recording of life limiting conditions across financial year of death (Table S1), with no clear trend over time.

Cause of death by age at death

In Scotland, recording of a life limiting condition as underlying cause of death was lowest (239, 41%) amongst under 1 year olds, but they had the highest percentage of life limiting condition-related deaths (150, 26%) (Table S2). Under 1 and over 20 year olds were most likely to have no life limiting condition among any cause of death (under 1s: 19%; 21-25 year olds: 21%; over 25 year olds: 19%).

Table S2: Recorded cause of death for cohort members, split by age group at death.

Life limiting condition recording		Age at death: Under 1 year	1-5 years	6-10 years	11-15 years	16-20 years	21-25 years	> 25 years	All ages
As underlying cause	Matched*	239 (41%)	185 (60%)	114 (67%)	147 (70%)	236 (62%)	264 (63%)	106 (59%)	1,291 (57%)
	Related*	150 (26%)	51 (17%)	20 (12%)	28 (13%)	36 (10%)	23 (6%)	11 (6%)	319 (14%)
**As contributing cause**		84 (14%)	30 (10%)	15 (9%)	18 (9%)	49 (13%)	44 (11%)	28 (16%)	268 (12%)
With trauma-related underlying cause		*≤5*	*≤5*	*≤5*	*≤5*	*≤5*	*7*	*≤5*	*10*
**Not recorded**		113 (19%)	41 (13%)	22 (13%)	18 (9%)	57 (15%)	86 (21%)	34 (19%)	371 (16%)
**All deaths in age group**		586	307	171	211	378	417	179	2,249

*'Matched' underlying causes are those that matched diagnostic codes within the life limiting condition coding framework. 'Related' causes were considered to be related to framework diagnoses present for the individual in the inpatient data.

Cause of death by ethnic group

In Scotland, White individuals were more likely to have life limiting conditions recorded as underlying cause of death (675, 58%) than those in the South Asian (21, 49%) or Other (≤10, <50%) groups. Disclosure control prevented release of most values for non-White groups.

Table S3: Recorded cause of death for Scotland cohort members, split by ethnic group.

Life limiting condition recording		White	Ethnic group<br>South Asian	Other
As underlying cause	Matched*	675 (58%)	21 (49%)	≤10
	Related*	177 (15%)	≤10	≤10
As contributing cause		134 (11%)	≤10	≤10
With trauma-related underlying cause		≤10	≤10	≤10
Not recorded		184 (16%)	≤10	≤10
All deaths associated with ethnic group		1170	43	18

*'Matched' underlying causes are those that matched diagnostic codes within the life limiting condition coding framework. 'Related' causes were considered to be related to framework diagnoses present for the individual in the inpatient data.

Cause of death by deprivation category

In Scotland, recording of a life limiting condition as the underlying cause of death was less likely for those in the more deprived categories than in the least deprived categories (category 1: 50.4%, 95% CI 46.5-54.3%; category 5: 61.2%, 95%CI 56.0-66.4%).

Table S4: Recorded cause of death for Scotland cohort members, split by deprivation category of last recorded deprivation score. The categories are population weighted so that 20% of the general population is in each category.

Life limiting condition recording		Deprivation category<br>1 (most deprived)	2	3	4	5 (least deprived)
As underlying cause	Matched	312 (50%)	286 (57%)	245 (59%)	236 (64%)	205 (61%)
	Related	101 (16%)	70 (14%)	57 (14%)	42 (11%)	49 (15%)
As contributing cause		81 (13%)	61 (12%)	54 (13%)	39 (11%)	30 (9%)
With trauma-related underlying cause		≤10	≤10	≤10	≤10	≤10
Not recorded		125 (20%)	86 (17%)	59 (14%)	49 (13%)	51 (15%)
All deaths associated with deprivation category		619	503	415	366	335

* ‘Matched’ underlying causes are those that matched diagnostic codes within the life limiting condition coding framework. ‘Related’ causes were considered to be related to framework diagnoses present for the individual in the inpatient data.

Cause of death by diagnostic group

In Scotland, 94% of individuals with a haematology or oncology diagnosis had a life limiting condition as underlying cause of death; only 2% had no indication of life limiting conditions in their death records (Table S5). Only 36% of patients with a genitourinary diagnosis had a life limiting condition as the underlying cause of death and 33% no life limiting condition among any cause of death.

Table S5: Recorded cause of death for Scotland cohort members split by diagnostic group.

		Diagnostic group – Scottish data								
**Life limiting condition recording**		**Neurology**	**Haematology & Oncology**	**Respiratory**	**Circulatory**	**Gastrointestinal**	**Genitourinary**	**Perinatal**	**Congenital**	**Metabolic & Other**
**As underlying cause**	**Matched***	222 (47%)	900 (94%)	265 (56%)	93 (54%)	59 (43%)	104 (36%)	91 (34%)	254 (46%)	183 (79%)
	**Related***	106 (22%)	8 (1%)	60 (13%)	25 (15%)	17 (12%)	28 (10%)	72 (27%)	155 (28%)	10 (4%)
**As contributing cause**		75 (16%)	30 (3%)	75 (16%)	28 (16%)	35 (25%)	63 (22%)	50 (19%)	86 (15%)	17 (7%)
With trauma-related underlying cause		*≤5*	*≤5*	*≤5*	*≤5*	*7*	*≤5*	*≤5*	*≤5*	*≤5*
**Not recorded**		74 (16%)	23 (2%)	75 (16%)	26 (15%)	27 (20%)	96 (33%)	53 (20%)	60 (11%)	21 (9%)
**All deaths associated with diagnostic group**		477	961	475	172	138	291	266	555	231

* ‘Matched’ underlying causes are those that matched diagnostic codes within the life limiting condition coding framework. ‘Related’ causes were considered to be related to framework diagnoses present for the individual in the inpatient data.

Multivariable model

The multivariable model for the Scottish data (Table S6) showed no significant variations by age.

Neither were there significant variations by ethnic group or deprivation category (although for deprivation category there was an apparent trend towards increased likelihood of life limiting condition recording for individuals from less deprived areas, albeit not significant). Primary diagnostic group showed similar patterns to those seen in the univariable analyses: haematology

and oncology diagnoses were most likely to be associated with life limiting condition recording in death records and genitourinary, gastrointestinal and perinatal diagnoses least likely.

Table S6: Logistic regression model, using the Scottish data, for the odds ratio of a death record containing any indication of life limiting conditions (underlying, related or contributing) depending on age group at death, ethnic group, deprivation category and primary diagnostic group.

	Odds Ratio for life limiting conditions in death record	95% confidence interval		P value
**Age group**				
Under 1	1 (ref)			
1-5	0.95	0.53	1.68	0.85
6-10	0.84	0.42	1.70	0.63
11-15	1.11	0.52	2.36	0.79
16-20	0.88	0.49	1.57	0.67
21-25	0.58	0.32	1.05	0.07
> 25	0.73	0.36	1.48	0.38
**Ethnic group**				
White	1 (ref)			
South Asian	1.44	0.57	3.66	0.45
Other	0.63	0.21	1.89	0.41
**Last recorded deprivation category**				
1 (most deprived)	1 (ref)			
2	1.06	0.67	1.65	0.81
3	1.15	0.71	1.86	0.58
4	1.27	0.74	2.19	0.39
5 (least deprived)	1.40	0.79	2.49	0.26
**Primary diagnostic group**				
Neurology	0.09	0.04	0.19	< 0.01
Haematology and oncology	1 (ref)			
Respiratory	0.06	0.03	0.14	< 0.01
Circulatory	0.08	0.03	0.20	< 0.01
Gastrointestinal	0.05	0.02	0.13	< 0.01
Genitourinary	0.02	0.01	0.05	< 0.01
Perinatal	0.06	0.02	0.18	< 0.01
Congenital	0.17	0.08	0.39	< 0.01
Metabolic and Other	0.14	0.05	0.43	< 0.01
**Model characteristics**				
Log likelihood	-452			
BIC	1053			
Degrees of freedom	21			

REFERENCES

1. ISD Scotland. Electronic Data Research and Innovation Service (eDRIS). http://www.isdscotland.org/Products-and-Services/EDRIS/.
2. Buchanan D. *ISD Statistical Disclosure Control Protocol*. Edinburgh: ISD Scotland Publications, 2009.
3. Kendrick S and Clarke J. The Scottish record linkage system. *Health bulletin*. Edinburgh1993, p. 72-9.
4. Kendrick S. The Development of Record Linkage in Scotland: The Responsive Application of Probability Matching. In: Alvey W and Jamerson B, (eds.). *Record linkage techniques – 1997 Proceedings of an International Workshop and Exposition*. Washington DC: Federal Committee on Statistical Methodology, Office of Management and Budget, 1997, p. 319-32.
5. Schwarz G. Estimating the dimension of a model. *The annals of statistics*. 1978; 6: 461-4.
6. Kass RE and Raftery AE. Bayes factors. *Journal of the American Statistical Association*. 1995; 90: 773-95.

Supplement Part 2: Sensitivity analyses for England

Table S7: Logistic regression model, using the English data, for the odds ratio of a death record containing any indication of life limiting conditions (underlying, related or contributing) depending on age group at death, ethnic group, deprivation category and primary diagnostic group. Sensitivity analysis for deaths from 1 April 2009 onwards when there are fewer missing ethnicity data.

	Odds Ratio for life limiting conditions in death record	95% confidence interval		P value
**Age group at death**				
Neonate	0.55	0.48	0.63	< 0.01
Post neonatal infant	1 (ref)			
1-5	0.71	0.60	0.83	< 0.01
6-10	0.67	0.55	0.83	< 0.01
11-15	1.03	0.83	1.29	0.76
16-20	0.69	0.58	0.82	< 0.01
21-25	0.55	0.47	0.64	< 0.01
> 25	0.71	0.60	0.84	< 0.01
**Ethnic group**				
White	1 (ref)			
Indian	1.11	0.87	1.41	0.39
Pakistani	1.51	1.28	1.78	< 0.01
Bangladeshi	1.34	0.96	1.86	0.09
Black	1.04	0.88	1.23	0.64
Mixed	1.13	0.89	1.45	0.31
Other	1.44	1.18	1.75	< 0.01
**Last recorded deprivation category**				
1 (most deprived)	1 (ref)			
2	1.11	1.00	1.24	0.06
3	1.21	1.07	1.36	< 0.01
4	1.19	1.04	1.36	0.01
5 (least deprived)	1.20	1.05	1.38	< 0.01
**Primary diagnostic group**				
Neurology	0.09	0.07	0.11	< 0.01
Haematology	0.06	0.05	0.08	< 0.01
Oncology	1 (ref)			
Respiratory	0.05	0.04	0.07	< 0.01
Circulatory	0.09	0.07	0.12	< 0.01
Gastrointestinal	0.03	0.02	0.04	< 0.01
Genitourinary	0.03	0.02	0.03	< 0.01
Perinatal	0.05	0.04	0.06	< 0.01
Congenital	0.10	0.08	0.12	< 0.01
Metabolic	0.18	0.13	0.24	< 0.01
Other	0.06	0.03	0.12	< 0.01
**Model characteristics**				
Log likelihood	-7048			
BIC	14366			
Degrees of freedom	28			

Table S8: Logistic regression model, using the English data, for the odds ratio of a death record containing any indication of life limiting condition(underlying, related or contributing) depending on age group at death, ethnic group, deprivation category and primary diagnostic group. Sensitivity analysis excluding neonates.

	Odds Ratio for a life limiting condition in death record	95% confidence interval		P value
**Age group at death**				
Post neonatal infant	1 (ref)			
1-5	0.76	0.68	0.85	< 0.01
6-10	0.76	0.66	0.88	< 0.01
11-15	1.00	0.86	1.16	0.98
16-20	0.82	0.72	0.93	< 0.01
21-25	0.63	0.56	0.71	< 0.01
> 25	0.76	0.67	0.87	< 0.01
**Ethnic group**				
White	1 (ref)			
Indian	1.07	0.88	1.31	0.49
Pakistani	1.36	1.19	1.54	< 0.01
Bangladeshi	1.14	0.87	1.49	0.34
Black	0.92	0.81	1.06	0.27
Mixed	1.10	0.87	1.39	0.42
Other	1.20	1.02	1.41	0.03
**Last recorded deprivation category**				
1 (most deprived)	1 (ref)			
2	1.09	1.00	1.20	0.06
3	1.20	1.08	1.32	< 0.01
4	1.23	1.10	1.37	< 0.01
5 (least deprived)	1.27	1.13	1.42	< 0.01
**Primary diagnostic group**				
Neurology	0.09	0.08	0.10	< 0.01
Haematology	0.06	0.05	0.08	< 0.01
Oncology	1 (ref)			
Respiratory	0.06	0.05	0.07	< 0.01
Circulatory	0.10	0.08	0.12	< 0.01
Gastrointestinal	0.03	0.02	0.04	< 0.01
Genitourinary	0.03	0.02	0.03	< 0.01
Perinatal	0.05	0.04	0.06	< 0.01
Congenital	0.10	0.08	0.11	< 0.01
Metabolic	0.19	0.15	0.23	< 0.01
Other	0.08	0.05	0.13	< 0.01
**Model characteristics**				
Log likelihood	-10801			
BIC	21876			
Degrees of freedom	27			
